# A network analysis of statistics anxiety symptoms and their antecedents in UK higher education students

**DOI:** 10.1111/nyas.15350

**Published:** 2025-04-27

**Authors:** Joshua J. March, David Hamilton, Dawn McCormack, Ross Brisco, Amy Grech

**Affiliations:** ^1^ Department of Psychological Sciences and Health University of Strathclyde Glasgow UK; ^2^ Design, Manufacturing and Engineering Management University of Strathclyde Glasgow UK

**Keywords:** network analysis, peer attitudes, statistics anxiety

## Abstract

Statistics anxiety is a widespread, multifaceted phenomenon affecting many students in higher education. Feelings of excessive worry when exposed to statistical content impact student performance and heighten negative perceptions of statistics. While many factors have been identified as relevant antecedents of statistics anxiety, it is unclear how they relate to different components of this phenomenon, and which factors are most influential. Additionally, no research has investigated the impact of peer attitudes toward statistics anxiety. The current study describes a preregistered network analysis of statistics anxiety, peer attitudes, and related variables with a sample of 279 UK higher education students. After performing reliability checks, results support the distinction made in previous literature between attitudes toward statistics and statistics anxiety per se. The former were influenced by feelings of statistics self‐efficacy, age, and peer attitudes toward statistics, and the latter was influenced by negative problem orientation and intolerance of uncertainty. The most influential nodes were the negative problem orientation variables, inhibitory anxiety, and interpretation anxiety. The findings are discussed in relation to addressing statistics anxiety from multiple angles.

## Graphical abstract

The current study performs a network analysis with 279 UK higher education students to examine the relationship between statistics anxiety and various psychological antecedents identified in previous research. The generated network suggests that attitudes toward statistics and statistics anxiety per se are impacted by different variables. The findings are discussed in the context of addressing statistics anxiety in education.

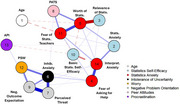

## INTRODUCTION

Statistical literacy, the ability to interpret and assess statistical information, is a core component in psychology programs—yet no other topic causes students more anxiety and worry.^1^ Estimates of the prevalence of statistics anxiety (SA) vary, but most studies agree that it affects a sizeable subset of students. Onwuegbuzie^2^ states that 60%–80% of graduate students experience uncomfortable levels of SA, but this was based on a small sample of 135 graduate students, and the cutoffs for “uncomfortable” were based on percentile rank norms from an older paper.^3^ More recently, Abdalla^4^ found that 71% of 142 social work/sociology students experienced SA—another study identified 27% of 440 social science students as having SA.^5^ While the exact prevalence is unknown, the above figures suggest that SA affects a significant proportion of students. Given that SA can negatively impact statistics performance^6^, ^7^ (but see Ref. 8), investigating the antecedents of SA is a crucial research area.

### Statistics anxiety

As with estimates of its prevalence, there are differing definitions of SA. Zeidner^9^ defined SA as “excessive worry, intrusive thought or tension occurring when exposed to statistical content.” Onwuegbuzie and Wilson^1^ suggested “a negative emotional state encompassing behavioral and cognitive symptoms that commence when the individual is faced with environments, tasks or evaluations related to statistics.” However, Papousek et al.^10^ note that definitions like these conflate SA and *attitudes toward statistics*—the former is the negative reaction experienced when confronted with statistics, but the latter is the value judgment held toward statistics themselves. Chew and Dillon^11^ thus offer the following definition: “A negative state of emotional arousal experienced by individuals as a result of encountering statistics in any form and at any level; this emotional state is preceded by negative attitudes toward statistics and is related to but distinct from mathematics anxiety.” This distinguishes SA from mathematics anxiety and from the antecedents leading up to it.

Assessments of SA can also conflate SA and attitudes toward statistics, yet they usually agree that SA is a multifaceted construct. Most widely used is the Statistics Anxiety Rating (STAR^3^) scale, which has a six‐factor structure: Test and Class Anxiety, Interpretation Anxiety, Fear of Asking for Help, Worth of Statistics, Fear of Statistics Teachers, and Computational Self‐Concept. The former three items are argued to assess SA, and the latter three assess attitudes toward statistics,^10^, ^12^ matching Chew and Dillon's^11^ distinction of these concepts. The STAR is demonstrated to be reliable with the six‐factor structure consistently validated, with different subscales found to be differently influential.^11^, ^12^, ^13^, ^14^ Given the multifaceted nature of SA, it is therefore unsurprising that SA is related to many different antecedents and outcomes.

Many factors have been identified as relevant antecedents of SA, predisposing or heightening students’ negative experiences with, and attitudes toward, statistics. Onwuegbuzie and Wilson^1^ distinguish three categories of SA antecedents: situational (prior knowledge of statistics; statistics course grades…), dispositional (perceived mathematics self‐concept; self‐esteem…), and environmental (gender differences; age…). Cui et al.^15^ further divided dispositional antecedents into three subcategories: demographic factors; factors relating to the learner (personality variables, perceived value of statistics tasks); and factors relating to behavior (e.g., procrastination, learning strategies). Supporting this, a recent review and meta‐analysis by Trassi et al.^7^ found that procrastination and self‐efficacy showed strong relationships with SA, whereas the evidence for negative affect sociodemographic factors and learning strategies was unclear. Additionally, both SA and antecedents of SA can have direct/indirect impacts on statistics performance.^6^, ^16^

### Peer attitudes toward statistics

Given that attitudes toward statistics are associated with SA, it is plausible that perceptions of *others’* attitudes toward statistics can impact SA. Peer groups impact academic achievement, beginning young and increasing into adolescence.^17^ A plausible pathway for peer attitudes to impact SA would be via self‐efficacy, as this impacts SA and could be impacted by perceptions of peer attitudes.^18^ This in turn may increase positive experiences of statistics, improving intrinsic motivation and cooperative learning.^19^ The evidence for the relationship between peer attitudes and SA is currently mixed and scarce. Diaconu‐Gherasim et al.^20^ found that while peer cohesiveness and cooperation was related to students’ self‐efficacy, the relationship was not significant once teacher support was accounted for, arguing that teachers, not peers, have a particularly powerful role in promoting self‐efficacy. In contrast, Llorca et al.^18^ found that adolescent peer relationships were related to academic self‐efficacy, with peer attachment promoting self‐efficacy and victimization weakening it.

Looking at developmental research, there is again limited evidence that peer attitudes matter. Quane^21^ observed that young children's engagement with mathematics was strongly impacted by their peers’ attitudes, and positive social support helped engagement. Mata et al.^19^ found that perceived peer support predicted attitudes toward mathematics in children and adolescents. Peer support was evaluated using the “In my Math class” scale^22^, ^23^ with questions like “In math class students want me to do my best in math work.” This suggests that perceptions of peer attitudes toward math impact student engagement. Furthermore, Fraser and Kahle^24^ found that perceptions of peer attitudes toward science predicted middle schoolchildren's pro‐science attitudes. However, the classroom environment, that is, perceptions of high‐quality teaching, was a stronger predictor of attitudes and achievement. This again mirrors the findings from Diaconu‐Gherasim et al.^20^ in that teachers have a stronger impact than peers. Given the mixed evidence and the lack of direct investigations into peer attitudes and SA, one aim of the current paper is to provide an initial assessment of peer attitudes toward statistics and its relationship with SA.

### Psychological network analysis

One way of identifying the impact of variables like self‐efficacy, negative affect, and peer attitudes on SA is through network analysis.^25^, ^26^ Psychological networks were developed to model connections between individuals but have since been used to examine relationships between variables, particularly in mental health research. By treating mental disorders as networks of symptoms that influence each other, it becomes possible to identify and target the most influential symptoms for treatment.^27^ In network analysis, each symptom (e.g., a questionnaire item, or subscale total) is represented by a node, and the relationships between nodes are represented by edges. These edges can be directed (e.g., if symptom A causes symptom B), undirected (if A and B are just related), and weighted to model the connection's strength.

Psychological network analyses have become widespread, with many tutorials available.^28^ However, best practice for such analyses is not always endorsed, causing interpretation problems. As network analysis usually provides an image of the nodes and edges, readers may overly rely on these visual estimates where spacing is not always meaningful. For example, the default “spring” setting in the popular network analysis R package *graph* ^29^ places the nodes to minimize distance between them, but this is merely cosmetic. Furthermore, any network estimated from a dataset, particularly with small samples, may be unreliable and affected by small changes to participant numbers. To be confident in the position and relationship between nodes, it is highly recommended to calculate indicators of a network's reliability.^26^, ^28^, ^30^

We are aware of only two network analyses examining SA. Siew et al.^31^ examined the structure of the STAR in 228 US students with community analysis, finding distinct nodes of importance for participants with high versus low SA. For example, perceptions of statistics teachers as abstract impacted high anxiety students but not low anxiety students. Additionally, low anxiety students felt more strongly that statistics were worthless. The authors argue that students with high SA are more affected by negative internal attributions (e.g., self‐doubt about one's statistics competence) and low SA students are affected more by negative external attributions (e.g., statistics is not relevant). There are however reasons to treat these results with caution.^32^ First, Siew et al.^31^ state that their sample of 228 is comparable to previous studies. While this may be true, they actually divided their sample into two groups, the high SA group (*n* = 115) and the low SA group (*n* = 113), performing separate analyses on each. And although the authors calculate indicators of node centrality (strength, betweenness, and closeness), these estimates were not evaluated using bootstrapping methods.^26^, ^28^ Assessing the reliability of a network is a critical step given the potential variability of these estimates.

More recently, Huang et al.^33^ conducted a network analysis on SA with 1607 Chinese students—specifically, they employed latent profile analysis to divide their sample into three groups, mild anxiety (*n* = 802), moderate anxiety (*n* = 662), and high anxiety (*n* = 143), before estimating networks for these separate groups. The authors argue, similarly to Siew et al.,^31^ that students with differing levels of SA showed different influential symptoms, for example, concerns about making decisions in statistical analyses were more prevalent in the medium and high SA groups, but not the mild SA group. However, once again there are limitations with this study. Huang et al.^33^ specify they performed bootstrapping to evaluate the stability of the network centrality estimates and the correlation stability coefficient values. Such steps are best practice, but these results are not provided in the main paper nor on any supplemental material we could find online. As such it is still unclear how reliable these results are. As the sample size varies between the three networks (from *n* = 802 to *n* = 143), it would have been helpful to see if centrality stability also varied.

### The present study

The aim of the present study is to expand on previous work by performing a preregistered, exploratory network analysis of SA and its antecedents to determine which factors are most influential in higher education students. The novelty of the paper comes from two aspects: first, the paper includes assessments of SA as well as crucial variables identified by the meta‐analysis performed by Trassi et al.^7^ Rather than perform network analysis on the item level on the STAR, we included assessments of procrastination, statistics self‐efficacy, and negative affect to identify the relationships between components of the STAR and these variables. Second, we included an assessment of peer attitudes toward statistics to determine how it impacts SA. We conducted reliability analyses to check the accuracy of the estimated network, and we have made the data fully available.

## Methods

### Participants

The study was preregistered on the AsPredicted repository in November 2023 (https://aspredicted.org/D62_BBH). All materials and data can be found on its associated ResearchBox #2274 (https://researchbox.org/2274). Data collection was conducted between November 2023 and January 2025 via Qualtrics XM (Qualtrics^34^). The survey was circulated on a Scottish University's online research participation pool, Sona systems (https://www.sona‐systems.com/), and on social media. University undergraduates participated for research credits. Participation was limited to higher education students, between 18 and 65 years of age, who had completed a research methods module as part of their degree program. Ethical approval was granted by the University of Strathclyde's Departmental Ethics Committee (approval code 05.12.10.2023.A).

The initial sample who completed the study was 284 participants. Following our preregistration guidelines, we removed one participant with >30% of missing data, three participants under 18 years, and one participant who showed no variation at all in their responses (i.e., was rote clicking through the study). This left our final sample of 279 participants (244 female, 27 male, 8 nonbinary/third gender) aged between 18 and 53 years (*M*_Age_ = 20.67, *SD*_Age_ = 3.9). See Table 1 for sample frequencies for ethnicity, degree program, program year, and mode of attendance. In our preregistration, we specified that for network analysis, Epskamp et al.^26^ recommend a minimum sample of 250—our sample meets this threshold.

**TABLE 1:** Sample frequencies for the final set of participants.

Year of study		Ethnicity		Program of study		Mode of attendance	
Year 1	29	Arabic	12	Biology	1	Full‐time	277
Year 2	130	Asian	16	Business	2	Part‐time	2
Year 3	102	Black	2	Education	15		
Year 4	17	Other	10	Humanities	15		
Postgraduate	1	White	239	Languages	1		
				Law	2		
				Psychology	243		

### Measures

Our study included variables identified by Trassi et al.^7^ as key covariates of SA. Additionally, we produced a novel assessment of peer attitudes toward statistics based on the STAR.^3^ The following questionnaires were administered to the participants.

#### Demographics

Participants provided self‐report data for gender, ethnicity, age, year of study, program of study, and mode of attendance.

#### The Self‐Efficacy for Learning Statistics for Psychologists Scale

The Self‐Efficacy for Learning Statistics for Psychologists (SES‐Psy^35^) scale is a 40‐item questionnaire assessing students’ feelings of self‐efficacy for learning statistical techniques and concepts. Items are loaded onto four factors: self‐efficacy for basic statistics (e.g., “I can recognize the scale level [i.e., nominal, ordinal, interval] of a variable”), for advanced statistics (e.g., “I am happy to read and understand any book on statistics”), SA (reverse‐coded, e.g., “Before the statistics exam I was more upset than most of my colleagues”), and perceived relevance of statistics (e.g., “Statistics are important for scientists but not for me”). Participants respond on a six‐point Likert scale from 1 (not at all) to 6 (fully). Internal reliability for the full questionnaire was excellent, Cronbach's *α* = 0.91. Looking at the four subscales, reliability was 0.73 for both SA and perceived relevance of statistics, and 0.88 for self‐efficacy for basic statistics and 0.83 for self‐efficacy for advanced statistics. All subscales thus demonstrated acceptable internal reliability.

#### The Aitken Procrastination Inventory

The Aitken Procrastination Inventory (API^36^) is a 19‐item questionnaire assessing levels of procrastination in academic environments. Participants were asked to respond on a five‐item scale from 1 (false) to 5 (true). Internal reliability was excellent, Cronbach's *α* = 0.89.

#### The Procrastination Assessment Scale

The Procrastination Assessment Scale assesses students’ tendencies to procrastinate in six areas: writing an essay, studying for exam, keeping up with weekly reading, academic administrative tasks, academic attendance tasks, and university activities in general.^37^ For each area, students are asked three questions: “To what extent do you procrastinate on this task?”, responding on a scale from 1 (never procrastinate) to 5 (always procrastinate); “To what extent is procrastination on this task a problem for you?”, responding from 1 (not at all a problem) to 5 (always a problem); and “To what extent do you want to decrease your tendency to procrastinate on this task?”, responding from 1 (do not want to decrease) to 5 (definitely want to decrease). Internal reliability was excellent, Cronbach's *α* = 0.90.^a^

#### The STAR scale

The STAR scale is one of the most widely used assessments of SA. The 51‐item questionnaire assesses six different dimensions: perceived worth of statistics, fear of asking for help, interpretation anxiety, test and class anxiety, computational self‐concept, and fear of statistics teachers. The initial 23 items assess feelings of anxiety for different aspects of learning and performing statistics, with responses from 1 (no anxiety) to 5 (strong anxiety)—the latter 28 items assess agreement with different statements about feelings toward statistics, from 1 (strongly disagree) to 5 (strongly agree). Internal reliability for the full scale was excellent, Cronbach's *α* = 0.96. Looking at the six subscales, alphas were 0.94 for perceived worth of statistics; 0.88 for fear of asking for help; 0.90 for interpretation anxiety; 0.87 for class and test anxiety; 0.87 for computational self‐concept; and 0.82 for fear of statistics teachers.

#### The Peer Attitudes Towards Statistics scale

The Peer Attitudes Towards Statistics (PATS) scale is a new assessment designed to assess students’ perceptions of a close friend's attitude toward statistics. Participants were asked to keep in mind the peer within their statistics course that they were closest to. They then responded to 19 questions inspired by the 28 agreement items from the STAR, assessing their peers’ attitudes toward statistics (see the ResearchBox for the items). The items were questions that easily could be modified to account for a peers’ attitudes toward statistics. Participants responded on a five‐point scale, ranging from 1 (strongly disagree) to 5 (strongly agree). Internal reliability for this initial scale was excellent, Cronbach's *α* = 0.88, and was explored further during the factor evaluation (see below).

#### The Penn State Worry Questionnaire

The Penn State Worry (PSW) 16‐item questionnaire assesses feelings of worry and negative attitudes toward events.^38^ Participants respond to questions on a five‐point scale from 1 (not at all typical) to 5 (very typical). The internal reliability of the questionnaire was excellent, Cronbach's *α* = 0.93.

#### The Intolerance of Uncertainty Scale 12

The Intolerance of Uncertainty Scale 12 (IoUS‐12) is a short‐form of the 27‐item Intolerance of Uncertainty Scale,^39^, ^40^ which has positive associations with worry, anxiety, and excessive perceptions of threat. The shorter version has 12 questions loading onto two factors, prospective anxiety and inhibitory anxiety, and correlates strongly with the original.^39^, ^41^ Participants respond on a five‐point scale from 1 (not at all characteristic of me) to 5 (entirely characteristic of me). Internal reliability of the full scale was excellent, Cronbach's *α* = 0.91. For the subscales, the reliability for prospective anxiety was 0.84 and for inhibitory anxiety was 0.89. Both subscales thus showed excellent internal reliability.

#### The Negative Problem Orientation Questionnaire

The Negative Problem Orientation Questionnaire (NPOQ) is a 12‐item questionnaire assessing negative problem orientation, defined as a set of beliefs about problems as a threat to well‐being, experiencing doubt about problem‐solving ability and being pessimistic about outcomes.^42^, ^43^ This variable correlates with worry, depression, and social anxiety.^44^ While initial papers found a unitary structure, recent work argues for a bifactor model of a general NPO factor versus a three‐factor structure of perceived threat, self‐inefficacy, and negative outcome expectancy.^45^ To identify more nuanced relationships with other variables, we calculated the latter three subscales. Participants respond to questions on a five‐point scale from 1 (not at all true of me) to 5 (extremely true of me). Internal reliability of the full scale was excellent, Cronbach's *α* = 0.95. Looking at the subscales, the internal reliability for perceived threat was 0.81, for self‐inefficacy was 0.90 and for negative outcome expectancy was 0.88. All subscales thus showed excellent reliability.

### Procedure

Participants accessed the survey online from the SONA website/social media advert. They read a participant information sheet and provided consent before accessing the survey. Participants then completed the above questionnaires in order (with an estimated completion time of 20–25 min). Afterward participants were provided with a debrief explaining the rationale as well as links to mental health support services given the nature of the questionnaires. Only participants who arrived at the debrief were included in the study.

### Data analysis

All data were formatted and analyzed in R version 4.4.0,^46^ with the R Studio IDE (also known as posit), version 4.2.764.^47^ The main R packages used for the principal components analysis, multiple imputation and network analysis were *Hmisc*, *DT*, *FactorMineR*, *Factoshiny*, *plotly*, *ggbiplot*, *factoextra*, *corrplot*, *ltm*, *miceadds*, *miceafter*, *qgraph*, *naniar*, *networktools*, *bootnet*, *network*, *huge*, and *mgm*. A full list of the packages used is included in the R scripts in the ResearchBox.

Looking at skewness values and histograms, all variables were normally distributed except for three—age, the STAR Test Anxiety subscale, and the PSW total score, which had skewness values of 4.48, −1.004, and −0.515. We therefore estimated the network by applying the nonparanormal transformation included in the estimateNetwork function from the *bootnet* package.^26^ This provides accurate network estimations even when applied to normally distributed data.^28^ Given the uneven distributions for our categorical variables of gender, ethnicity, year of study, program of study, and mode of attendance, we omitted these variables from the network analyses. This left 20 initial nodes: age, the 6 subscales for the STAR, the 4 subscales for the SES‐Psy, the PSW total, the 2 subscales for the IoUS‐12, the API total, the 3 subscales for the NPOQ, the Procrastination Assessment Scale total, and the PATS total.

### Evaluation of the PATS factor structure

First, as we used a novel assessment for the PATS scale, we performed principal components analysis to determine if we should use the total or divide it into subscales. We excluded 5 cases with missing data on the PATS scale from this analysis, so the following analyses were conducted on a sample of 274 participants.

The Kaiser‐Meyer‐Olkin (KMO) test for factor adequacy on the PATS data showed acceptable levels for performing factor analysis, overall measure of sampling adequacy (MSA) = 0.89. The Bartlett test was highly significant, *χ^2^* (171) = 2422.13, *p* < 0.001, indicating that the data were suitable for principal components analysis. Examining the initial scree plot suggested two potential factors that met Kaiser's rule (eigenvalue > 1), whereas parallel analysis suggested four potential factors. We therefore compared a two versus four factors structure. We applied a promax rotation as we assumed items and factors could be correlated. The factor analysis with four factors showed that while this was sufficient, *χ^2^* (101) = 205.03, *p* < 0.001, only the first three factors had more than three items with loadings above 0.4. Additionally, the latter two factors only had two to four items loaded onto them. In contrast, a two‐factor structure showed better fit, *χ^2^* (134) = 489.56, *p* < 0.001. The two‐factor structure accounted for 44% of the total variance, but the first factor accounted for 34% of the variance.

We then evaluated Cronbach's *α* for these two factors. Internal reliability for the first factor was excellent, *α* = 0.91, but the second had poor reliability, *α* = 0.59. Case‐dropping reliability analyses showed that removing item 9 increased factor 2's reliability to 0.64 but left only four items for this factor and two loadings less than or equal to 0.4. We therefore evaluated the fit of a factor structure with only one underlying factor, as the scree plot and reliability analyses indicated that the first factor seemed more important. The fit of the one‐factor structure was excellent, *χ^2^* (152) = 683.88, *p* < 0.001, with 14 items loading onto it. One item (Item 2) only loaded on to this factor at 0.3, so it was removed. This left one factor assessed with 13 items. Cronbach's *α* for this factor was excellent, *α* = 0.91, and accounted for 34% of the variance. We then checked the internal reliability for this same factor on the full data (*n* = 279), including participants with missing data on the PATS. Internal reliability remained excellent, *α* = 0.91. We thus used this factor as our assessment of peer attitudes toward statistics. The final 13 items included in our modified PATS measure are provided in Table 2.

**TABLE 2:** Items included in the final Peer Attitudes Towards Statistics scale.

Item number in the original scale	Item text
3	My peers do not enjoy statistics
4	My peers do not enjoy maths; therefore, they do not enjoy statistics
5	My peers find statistics to be useless
6	My peers do not want to learn statistics
7	My peers do not want to learn to like statistics
8	My peers feel statistics takes more time than it is worth
9	My peers feel statistics teachers come across as inhuman
10	My peers believe that statistics is for people who naturally lean toward maths
11	My peers believe statistics is a pain they could do without
12	My peers wish that statistics was removed from their academic program
13	My peers do not understand why someone in their field would need statistics
14	My peers feel like statistics teachers speak another language
19	The overall attitude of my peers toward statistics is negative

### Multiple imputation for missing data analysis

Of the 279 participants, 53 had missing data, ranging from 1 to 20 items (0.52%–10.6% of all variables). As network analysis requires complete cases, we used multiple imputation to estimate the missing data. Data were not missing at random, *p* = 0.0249, but the levels of missingness were minimal, with the amount of missing data on the 20 variables ranging between 0% and 3.58%. Under low to medium missingness, it is acceptable to impute missing data with multiple imputation with chained equations under missing at random assumptions.^48^ We therefore used the *mice* function^49^ to estimate 10 datasets with imputed missing values. All analyses were performed across the different datasets, with one dataset chosen at random for representativeness below.^50^, ^51^ All scripts for reproducing datasets/analyses are presented in the ResearchBox. As we are not combining the different datasets, we can identify the stability of the network created from the data.

### Node reduction

After replacing all missing values via multiple imputation, as per our preregistration, we checked for node redundancy using the *goldbricker* function.^52^ This examines whether nodes within a network are redundantly measuring the same construct (and are thus collinear). We set the threshold proportion of significantly different correlations to 0.5—furthermore, for ease of interpretation we used the best‐goldbricker method to identify the variable with most unique variance in variable pairs and only keep this one. After performing the node analysis on our initial 20 variables, the goldbricker function returned 14 variables (see Table 3). Across all imputed datasets, the analyses returned 14 nodes (see ResearchBox for the R script for calculating each dataset).

**TABLE 3:** Variables included in the generated network and their respective node numbers.

Node number	Variable name
1	Age
2	SES‐Psy Statistics Anxiety
3	SES‐Psy Perceived Relevance of Statistics
4	STAR Fear of Asking for Help
5	STAR Worth of Statistics
6	IoUS‐12 Inhibitory Anxiety
7	NPOQ Perceived Threat
8	PATS Modified Score
9	NPOQ Negative Outcome Expectation
10	SES‐Psy Competence for Basic Statistics
11	STAR Fear of Statistics Teachers
12	PSW Worry
13	API Procrastination
14	STAR Interpretation Anxiety

Abbreviations: API, Aitken Procrastination Inventory; IoUS‐12, Intolerance of Uncertainty Scale 12; NPOQ, Negative Problem Orientation Questionnaire; SES‐Psy, Self‐Efficacy for Learning Statistics for Psychologists; STAR, Statistics Anxiety Rating; PSW, Penn State Worry; PATS, Peer Attitudes Towards Statistics.

### Network estimation

We then estimated the network from the dataset with the remaining 14 nodes. As the remaining variables were continuous, we estimated a Gaussian graphical model (GGM) using the *bootnet* package.^26^ We used LASSO regularization to estimate the network structure, with a hypertuning parameter set to 0.5. The EBICglasso estimator was used as it is recommended for regularized GGM estimation with lower sample sizes.^28^

After estimating the network, centrality indices for node strength, closeness, and expected influence were estimated using the *qgraph* package.^29^ For those unfamiliar with these indicators, we describe them here. *Node strength* is calculated as the sum of the absolute value of all connections of a node relative to all other nodes, and can be interpreted as a sign of how strongly a node is directly connected to the others.^27^, ^53^ *Node closeness* quantifies the distance from a node to all other nodes via the indirect connections, calculated as the average shortest path between a given node and the remaining nodes.^27^ High values indicate a short average distance from one node to all other nodes, meaning that node is highly connected to other nodes in the network. Additionally, as some items are expected to negatively correlate with each other, we compute *expected influence* in addition to strength. This estimate assesses a node's influence on its’ immediate neighbors (i.e., the nodes with which it shares an edge). This is identical to node strength, except that expected influence retains the positive or negative value for an edge weight. This means it can be interpreted more meaningfully in networks with both positive and negative edges.^27^ We point out that several previous network analyses have also computed node betweenness, defined as the number of times a node lies on the shortest path between two other nodes.^27^ However, simulation work shows that this estimate displays the poorest reliability, so we omit it here.^28^

To verify the accuracy of the estimated network and following our pre‐registration, we performed nonparametric bootstrapping (*n* = 1000) to estimate edge‐weight accuracy.^26^ We also estimated centrality stability by performing case‐dropping bootstrapping (*n* = 1000) to estimate network models based on subsets of the data. Finally, we computed the CS‐coefficient for each of the networks, which is a quantifier of the proportion of cases which can be dropped to retain a 0.7 correlation with the original centrality (with 95% certainty^26^).

## RESULTS

**FIGURE 1:**
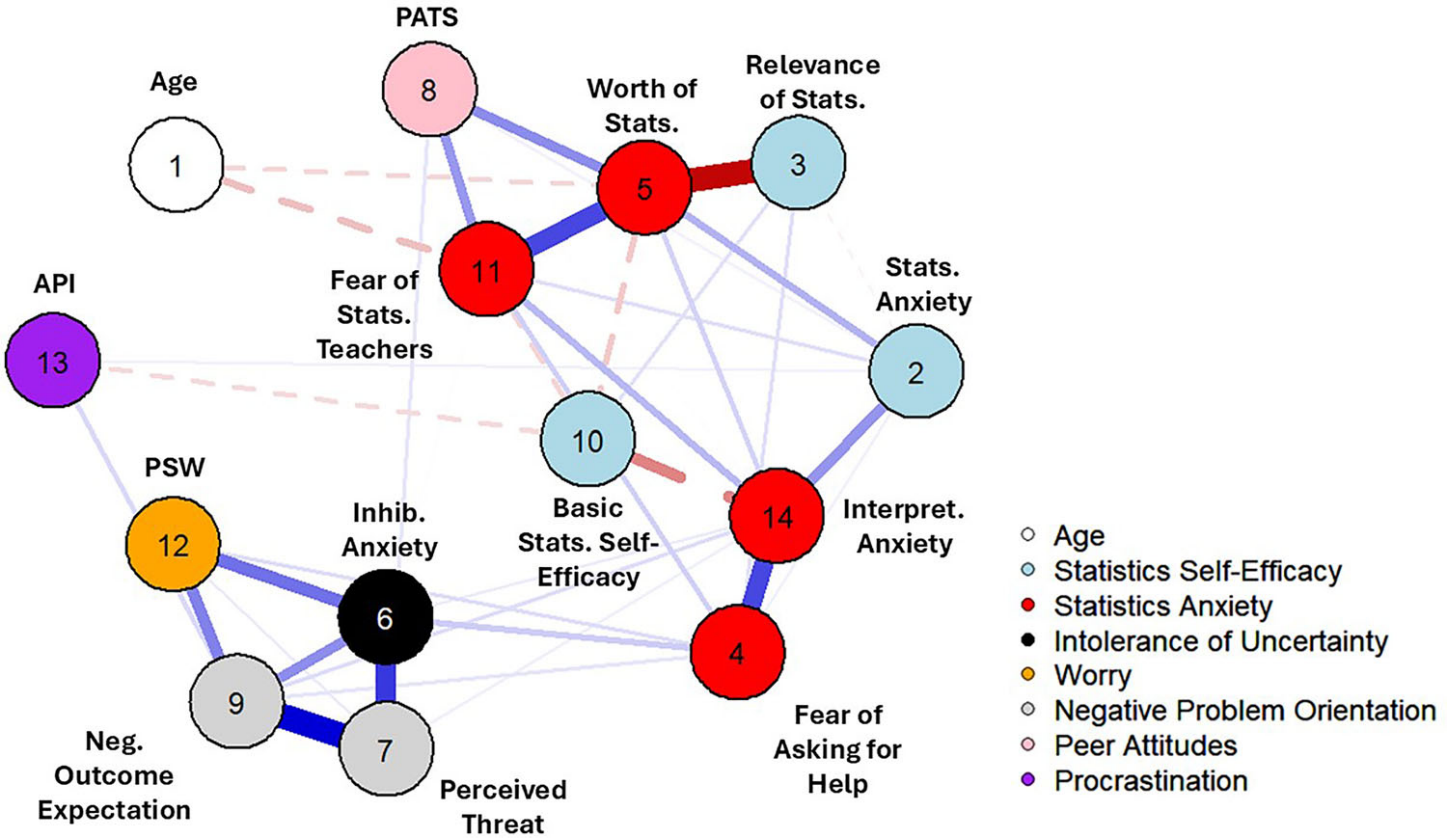
Network estimated from the dataset.

Figure 1 represents the network estimated from the dataset. All 10 imputed datasets, networks, and their analyses, along with those for the complete cases only data, can be generated from the R script in the ResearchBox. All node names have been included in Table 3. The subscales for worry, negative problem orientation, and intolerance of uncertainty (nodes 6, 7, 9, and 12) were highly correlated. This is expected given that these variables assess negative evaluations of uncertainty and future problems.^54^, ^55^ These negative affect subscales were also related to some SA nodes, in particular “Fear of Asking” for Help and Interpretation Anxiety. In contrast, other aspects of SA were more strongly related to statistics self‐efficacy—in particular, node 3 (relevance of statistics) and node 5 (worth of statistics) were highly negatively related, which is expected as the latter is negatively coded (higher scores mean less perceived value of statistics). Age (node 1) was negatively related to perceived worth of statistics (node 5) and fear of statistics teachers (node 11). Lack of perceived worth of statistics was also impacted by SA (node 2), peer attitudes toward statistics (node 8), and one's fear of statistics teachers (node 11). Fear of asking for help and interpretation anxiety were strongly related (nodes 4 and 14). Interpretation anxiety was impacted by SA (node 2), fear of asking for help (node 4), and negatively related to anxiety surrounding basic statistics (node 10). Finally, peer attitudes were positively related to both perceived worth of statistics and fear of statistics teachers.

### Edge‐weight stability bootstrapping

**FIGURE 2:**
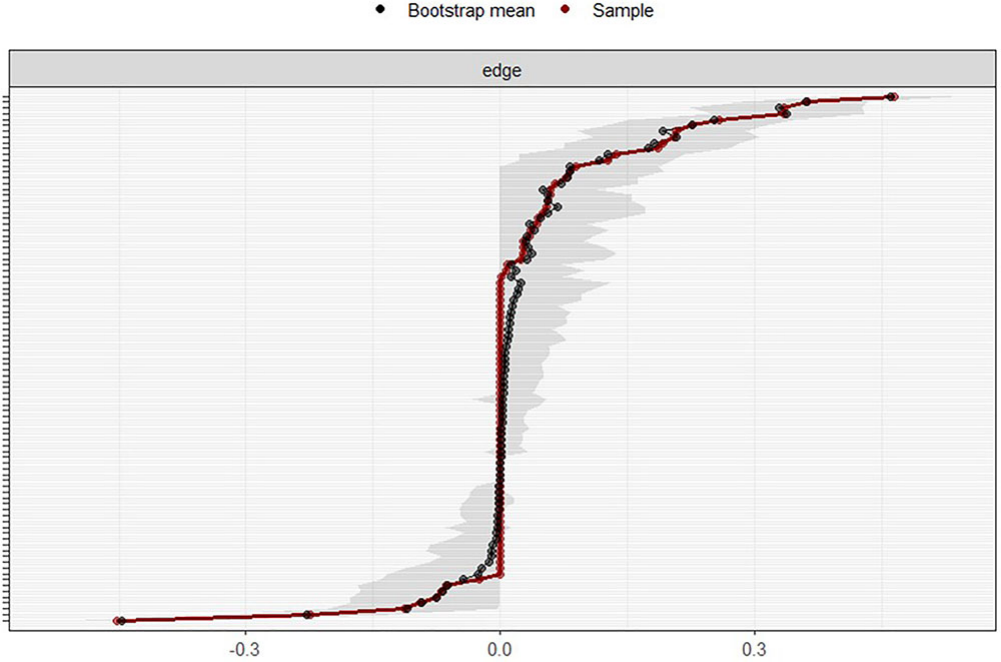
Bootstrapping (*n* = 1000) to estimate the stability of the edge‐weights for the estimated network.

We performed stability bootstrapping (*n* = 1000) to estimate the stability of the edge‐weights—Figure 2 shows that edge‐weight stability was reasonably accurate, remaining within the 95% CIs across bootstrapped samples.

### Centrality indices

**FIGURE 3:**
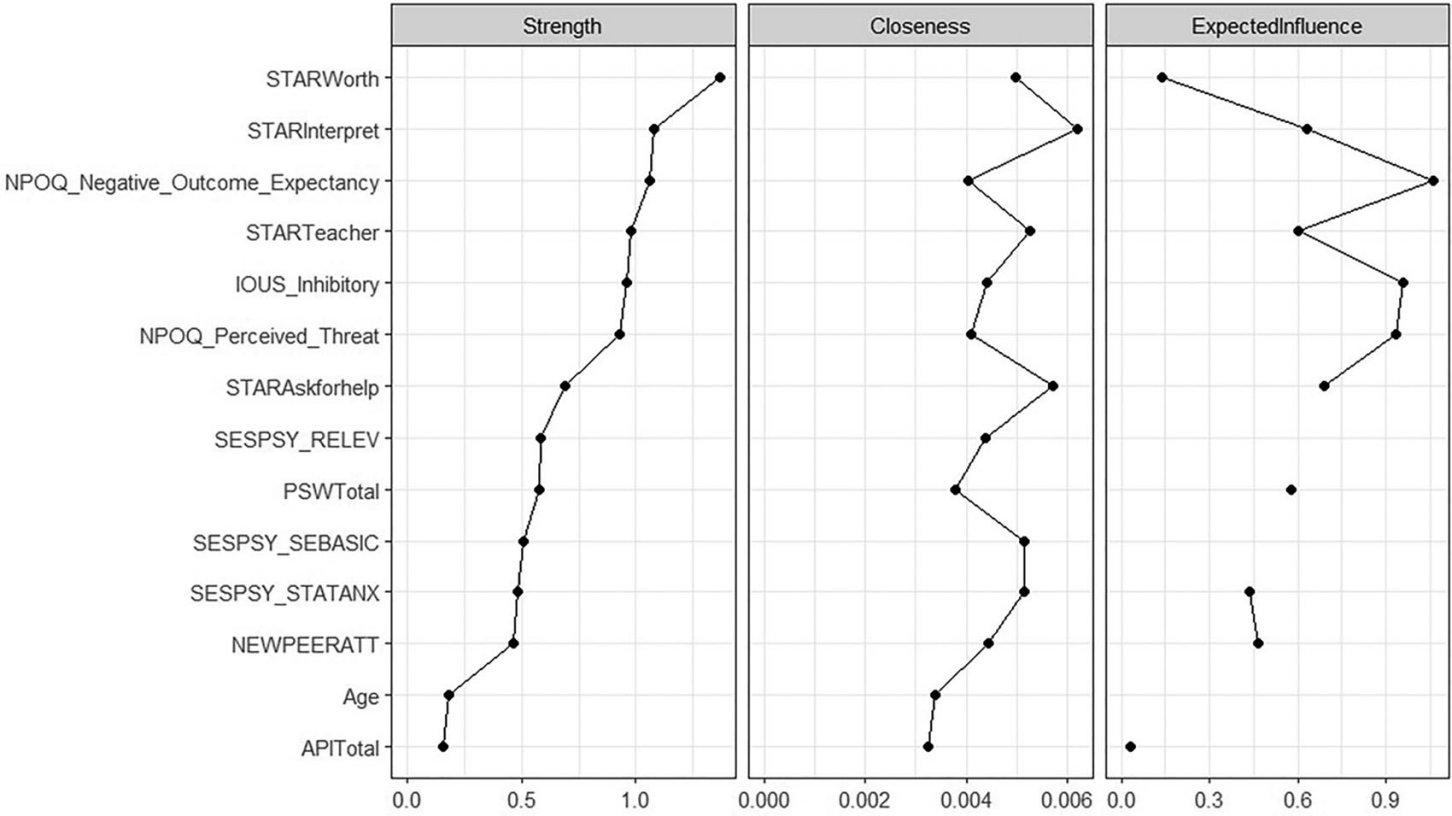
Estimates of strength, closeness, and expected influence calculated for all nodes in the dataset. Sorted in descending order for strength.

We computed centrality indices for strength, closeness, and expected influence (see Figure 3). The highest‐scoring nodes were the STAR “Perceived Worth of Statistics,” “Interpretation Anxiety,” “Fear of Statistics Teachers,” and “Fear of Asking for Help”; the NPOQ “Negative Outcome Expectancy” and “Perceived Threat”; and the IoUS‐12 “Inhibitory Anxiety” scales. While the STAR “Worth of Statistics” scored highest in strength, it was ranked much lower for expected influence (which is more reliable when networks contain positive and negative edges). In contrast, all other nodes were similarly the highest ranked for both strength and expected influence. Overall, this suggests that negative feelings about the future in general, different facets of SA, and attitudes toward statistics were most influential in the network.

### Case‐dropping bootstrapping for centrality stability

**FIGURE 4:**
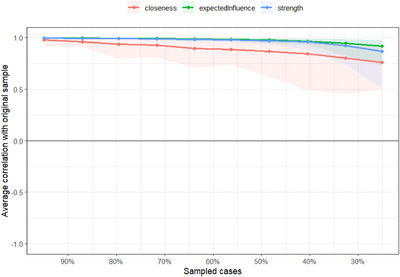
Case‐dropping bootstrapping to estimate the stability of the centrality estimates across different subsets of the data.

We performed case‐dropping bootstrapping to estimate the stability of the centrality indices across different subsamples of the data. As seen in Figure 4, the estimated correlations for strength and expected influence remained consistently high as the percentage of sampled cases increases.

### CS‐coefficient calculation

The estimate for strength was 0.674, the estimate for expected influence was 0.674, and closeness was 0.283. The CS‐coefficients across all 10 datasets can be seen in Table 4: the estimates for strength and expected influence remained consistent and all above 0.595, whereas the estimate for closeness varied between 0.129 and 0.437. This means we can be reasonably confident about our estimation for strength and expected influence, but caution should be taken when discussing interpretations of the closeness estimate.

**TABLE 4:** Centrality stability coefficients calculated for centrality estimates across the 10 imputed datasets’ networks.

	Network 1	Network 2	Network 3	Network 4	Network 5	Network 6	Network 7	Network 8	Network 9	Network 10
Strength	0.674	0.674	0.674	0.595	0.674	0.674	0.595	0.674	0.674	0.674
Closeness	0.437	0.204	0.283	0.283	0.283	0.283	0.362	0.129	0.204	0.129
Expected influence	0.749	0.674	0.674	0.595	0.674	0.674	0.595	0.674	0.674	0.674

### Consistency across imputed datasets

Table 5 presents the node estimates for strength and expected influence in the network, as well as the median values for all nodes present across the 10 imputed dataset networks. We omitted closeness given the low reliability for its CS‐coefficients. We also include the count for each node, that is, the number of networks in which that node remained after node reduction and network generation. The nodes consistently ranked in the top 50% of nodes across both measures were the NPOQ subscales (Perceived Threat and Negative Outcome Expectancy in particular), the IoUS‐12 Inhibitory Anxiety subscale, and several of the STAR subscales for “Worth of statistics,” “Fear of Asking for help,” “Interpretation anxiety,” and “Fear of Statistics Teachers.” The STAR subscale “Worth of Statistics” was consistently the strongest node but scored near the bottom for expected influence, so we caution against interpreting too much for it here. Additionally, all of these nodes remained after node reduction in all 10 of the networks, aside from “Fear of Statistics Teachers” (which appeared in 8 networks). This suggests we can be reasonably confident in their stability and their influence on the data. As argued above, the strongest and most influential nodes in all networks were ones assessing negative feelings about oneself and the future, and anxiety about interpreting and understanding statistical tests.

**TABLE 5:** Individual and median estimates for strength and expected influence for every node across the 10 imputed datasets. Count refers to the number of networks (out of 10) in which the variable appears after node reduction.

			Strength		Expected influence	
		Count	Network value	Median value	Network value	Median value
STAR	Worth of Statistics	10	1.37	1.359	0.137	0.117
	Interpretation Anxiety	10	1.081	1.067	0.633	0.646
	Fear of Asking for help	10	0.6897	0.675	0.6897	0.675
	Fear of Statistics Teachers	8	0.979	0.963	0.6029	0.614
	Computational Self‐Concept	2	n/a	0.8195	n/a	0.81
SES‐Psy	Relevance of Statistics	10	0.583	0.573	−0.372	−0.375
	Statistics Anxiety	10	0.486	0.493	0.436	0.436
	Fear of Basic Statistics	8	0.507	0.493	−0.407	−0.392
	Fear of Advanced Statistics	2	n/a	0.7805	n/a	−0.627
NPOQ	Perceived Threat	10	0.932	0.982	0.932	0.982
	Self‐Inefficiency	6	n/a	0.929	n/a	0.929
	Negative Outcome Expectancy	4	1.063	1.063	1.063	1.06
IoUS‐12	Inhibitory Anxiety	10	0.958	0.9865	0.958	0.9865
PSW	Worry	10	0.5782	0.58	0.5782	0.579
PATS	Peer Attitudes	10	0.465	0.4483	0.465	0.411
Age	Age	10	0.183	0.173	−0.183	−0.171
API	Procrastination	10	0.156	0.145	0.0284	0.016

Abbreviations: API, Aitken Procrastination Inventory; IoUS‐12, Intolerance of Uncertainty Scale 12; NPOQ, Negative Problem Orientation Questionnaire; SES‐Psy, Self‐Efficacy for Learning Statistics for Psychologists; STAR, Statistics Anxiety Rating; PSW, Penn State Worry; PATS, Peer Attitudes Towards Statistics.

## DISCUSSION

The present study aimed (a) to identify crucial symptoms in SA by performing a network analysis of SA and its antecedents;^7^ and (b) to explore the impact of peer attitudes toward statistics. Our network analysis showed that of the six STAR components, four consistently remained after node reduction: worth of statistics, interpretation anxiety, fear of asking for help, and fear of statistics teachers. These nodes showed distinct relationships with different antecedents of SA—fear of statistics teachers and worth of statistics were strongly related to perceived relevance of statistics, age, and peer attitudes, whereas fear of asking for help and interpretation anxiety were related to intolerance of uncertainty, SA, and poor self‐efficacy for basic statistics. This division matches distinctions from previous literature, as the former two STAR nodes assess attitudes toward statistics and the latter two assess SA proper.^11^ Our results provide further support for use of different STAR subscales for assessing distinct SA components.

With regards to the role of peer attitudes, our findings were mixed. Previous literature suggested that peer attitudes may impact SA via self‐efficacy,^18^ as positive peer attitudes toward statistics could affect self‐perceptions of competence. While the reduced PATS score remained after node reduction and showed distinct relationships with worth of statistics and perceived competence in advanced statistics, centrality assessments showed that the PATS only had limited influence on the network. There are several possible explanations: peer attitudes may just not be impactful on SA or attitudes toward statistics. A crucial factor affecting attitudes toward science and mathematics is teacher support—it could be the teacher, rather than peers, who impacts perceptions of self‐competence.^20^, ^24^ Another explanation is that our assessment of peer attitudes was not detailed enough. The PATS does not currently distinguish between peer attitudes and *agreement* with peer attitudes. Students who do not agree with the attitudes of their peers may not be influenced as much as students whose attitudes match their peers. As we did not assess congruence between self and peer attitudes toward statistics, this should be addressed by future research.

Indirect support for the importance of teachers in mitigating SA comes from our data—fear of statistics teachers as a node was relatively strong and retained across nearly all datasets. Fear of statistics teachers was positively related to worth of statistics (again negatively coded), interpretation anxiety, peer attitudes, and negatively related with age. This suggests that teachers have an important role to play in determining students’ perceptions toward statistics. Previous research suggests that age impacts attitudes toward statistics—older students are more positive about the usefulness of statistics.^56^, ^57^, ^58^ In our study too, age was associated with higher perceived worth of statistics, as well as reduced fear of statistics teachers. Furthermore, perceived worthlessness of statistics was strongly negatively associated with the SES‐Psy subscale of perceived relevance of statistics.^35^ These findings suggest that a key determinant of students’ attitudes toward SA is negative perceptions of statistics as a subject, informed by the attitudes of their peers and poor perceptions of statistics teachers.

Looking at the symptoms of SA proper, interpretation anxiety and fear of asking for help were consistent nodes across all imputed datasets and were consistently influential in all networks. Additionally, these nodes were related to intolerance of uncertainty, specifically the inhibition anxiety subscale, as well as to the NPOQ subscales and to feelings of worry. Interpretation anxiety was strongly associated with both the SES‐Psy SA and the STAR fear of asking for help subscales and negatively associated with the SES‐Psy self‐efficacy for basic statistics. This suggests that feelings of SA are impacted by negative attitudes toward seeking help, as well as low perceptions of one's own competence. For example, the STAR Fear of Asking for help subscale was impacted by the IoUS‐12 Inhibitory Anxiety scale—this is plausible given that the former deals with feelings of uncertainty around data, and the latter assesses avoidance of uncertainty. Viewing uncertain situations as threatening and being unable to cope with uncertainty may inhibit student engagement with statistics by provoking anxiety. Several factors may contribute to making learning statistics more opaque—a lack of clear explanations by the teacher; complicated GUIs with multiple options that must be clicked / unclicked; ill‐defined problems and learning outcomes…

Additionally, interpretation anxiety was impacted by fear for asking for help, but also by self‐efficacy. Previously, self‐efficacy for self‐regulated learning has been associated with help‐seeking behaviors—students with low levels of self‐efficacy avoid seeking help even though they need of it.^59^ Our findings extend this by showing that self‐inefficacy is related to stronger feelings of anxiety around interpreting statistical information, which is strongly related to fear of asking for help. Students with poorer perceptions of their own competence were more likely to feel anxious about interpreting statistical information. Negative perceptions of one's competence and the benefits of asking for help may strengthen interpretation anxiety, and vice‐versa: this is supported by the fact that the two SA symptoms were related to the final cluster in our network, the negative affect symptoms. The subscales of the NPOQ, along with IoUS‐12 Inhibitory Anxiety and worry assessed by the PSW, were all strongly related in our network across all datasets. This is not surprising as these assess various forms of negative affect and correlate strongly in previous literature.^42^, ^60^

The centrality checks showed that the Perceived Threat subscale for the NPOQ and the IoUS‐12 Inhibitory Anxiety subscale were particularly consistent and influential—this was consistent across all the imputed datasets and centrality indices. As mentioned above, these nodes showed relationships with the SA subscales of the STAR (interpretation anxiety and fear of asking for help). Lower tolerance of uncertainty, stronger expectations of threat in the future, and more self‐doubt over one's self‐efficacy led to more negative feelings about asking a teacher or peer for help in understanding statistics, and about interpreting statistical information.^45^ Therefore, a key factor impacting SA may be pessimistic views about asking for help—if students believe asking for help will make no difference, they will not be motivated to seek assistance. Students most in need of help seem less likely to seek it—schoolchildren with lower prior knowledge tend to show less help‐seeking behaviors than more knowledgeable peers.^61^ These results indicate that addressing students’ perceptions of the value of asking for help is a crucial step in reducing SA.

Based on our findings, we hypothesize that several key elements of SA could be targeted for intervention via different pathways. First, students’ attitudes toward statistics (i.e., fear of statistics teachers and perceived worth of statistics) are related to the perceived relevance of statistics, age, and peer attitudes toward statistics. Furthermore, fear of statistics teachers and perceived worth of statistics were strongly related to each other. Educators should therefore focus on promoting statistics as an exceedingly worthwhile task for everyone to engage in, not just statistically minded individuals. This seems especially important given the potential of statistics teachers to influence perceptions of the value of statistics, further highlighting the need for strong pastoral support in statistics education.^20^, ^24^ Second, interpretation anxiety was affected by self‐efficacy for basic statistics and negative problem expectations—this could be addressed by (a) encouraging students that they are capable of solving the task in question and (b) that asking for help will lead to a positive outcome and progress on the task. Promoting student questioning in an active, structured way^62^ may provide students with a more positive questioning experience. Student self‐efficacy is impacted by perceptions of teacher support,^20^, ^24^ meaning that an impactful change is partly within the control of teachers to SA implement. Third, anxiety surrounding asking for help was impacted by intolerance of uncertainty, worry, and negative outcome expectancy. Encouraging students to become more comfortable with uncertainty as a temporary state is predicted to reduce SA. Encouraging uncertainty tolerance is associated with increased emotional well‐being in medical contexts.^63^, ^64^ It therefore seems like SA teacher priorities could be clarifying/helping students manage uncertainty; promoting positive views of students’ own competence and self‐efficacy; and ensuring that statistics is framed as a worthwhile endeavor for everyone, not just “those who get it.”

The findings from the present study have several limitations. First, the sample was limited to higher education students in the United Kingdom, most of whom were enrolled in a psychology program. Statistics is employed in a wide variety of disciplines, and SA impacts students differently depending on their chosen discipline.^16^ Additionally, the sample was not very diverse in terms of gender or ethnicity, limiting the generalizability of the findings. Given previous mixed results regarding demographic factors like age, gender, and ethnicity,^1^, ^7^ future work should investigate the profile of SA symptoms across different populations, which is particularly important for promoting accessibility. A further limitation is the lack of information regarding statistics achievement. Recent studies have examined the relationships between SA and achievement, with mixed findings.^6^, ^13^, ^16^ Identifying the impact of different SA symptoms on achievement would be helpful to further strengthen the calls for targeting specific aspects of SA for support. Finally, our centrality assessments showed that while fear of asking for help, fear of statistics teachers, and interpretation anxiety were key network nodes across all measures and networks, perceived worth of statistics showed mixed results (very high in strength but low in expected influence). We therefore place more emphasis on the other factors as key symptoms of SA, until the role of worth of statistics can be clarified by future work.

In summary, this paper describes a preregistered network analysis of the STAR and relevant antecedents to SA identified by previous research. We also investigated the impact of peer attitudes on SA via a novel assessment. Replicating previous research, subscales of the STAR split into factors assessing SA and factors assessing attitudes toward statistics, and these factors were affected by different antecedents of SA. Fear of statistics teachers and worthlessness of statistics were strongly related to relevance of statistics, age, and peer attitudes, whereas fear of asking for help and interpretation anxiety were related to self‐efficacy for basic statistics, intolerance of uncertainty, worry, and negative outcome expectation. We found limited evidence for the role of peer attitudes toward aspects of attitudes toward SA, but further research is required to understand this influence. The above nodes were the most important ones identified in the network, suggesting that targeting these symptoms could help reduce/manage SA in higher education students.

## AUTHOR CONTRIBUTIONS

All authors contributed to the design of the study. Dawn McCormack designed the survey, collected data and performed preliminary analyses. Joshua J. March and David Hamilton provided feedback on the survey and also collected data. Joshua J. March submitted the pre‐registration, performed the analyses, wrote the manuscript and provided the open materials on ResearchBox. David Hamilton, Ross Brisco, and Amy Grech critically reviewed the manuscript.

## CONFLICT OF INTEREST STATEMENT

The authors declare no conflicts of interest.

### PEER REVIEW

The peer review history for this article is available at: https://publons.com/publon/10.1111/nyas.15350.

## Footnotes

a The original format of the Procrastination Assessment Scale involves summing together the results of the first two questions, but more recent versions include the summed total of the three questions. We investigated both versions and results are comparable.
